# *NOTCH2* Is Neither Rearranged nor Mutated in t(1;19) Positive Oligodendrogliomas

**DOI:** 10.1371/journal.pone.0004107

**Published:** 2009-01-01

**Authors:** Magdalena Benetkiewicz, Ahmed Idbaih, Pierre-Yves Cousin, Blandine Boisselier, Yannick Marie, Emmanuelle Crinière, Khê Hoang-Xuan, Jean-Yves Delattre, Marc Sanson, Olivier Delattre

**Affiliations:** 1 INSERM, Unité 830, Paris, France; 2 Institut Curie, Section Recherche, Unité de Génétique et Biologie des Cancers, Paris, France; 3 INSERM, Unité 711, Groupe hospitalier Pitié-Salpêtrière, Paris, France; 4 Université Pierre et Marie Curie-Paris 6, Laboratoire Biologie des Interactions Neurone-Glie, Groupe hospitalier Pitié-Salpêtrière, Paris, France; 5 AP-HP, Groupe hospitalier Pitié-Salpêtrière, Service de neurologie Mazarin, Paris, France; Texas Tech University Health Sciences Center, United States of America

## Abstract

The combined deletion of 1p and 19q chromosomal arms is frequent in oligodendrogliomas (OD) and has recently been shown to be mediated by an unbalanced t(1;19) translocation. Recent studies of 1p/19q co-deleted OD suggest that the *NOTCH2* gene is implicated in oligodendrocyte differentiation and may be involved in this rearrangement. The objective of the present study was to analyze the *NOTCH2* locus either as a chromosomal translocation locus that may be altered by the 1p/19q recurrent rearrangement or as a gene that may be inactivated by a two hit process. We performed an array-CGH analysis of 15 ODs presenting 1p/19q co-deletion using a high-density oligonucleotide microarray spanning 1p and 19q pericentromeric regions with 377 bp average probe spacing. We showed that the 1p deletion extends to the centromere of chromosome 1 and includes the entire *NOTCH2* gene. No internal rearrangement of this gene was observed. This strongly suggests that the t(1;19) translocation does not lead to an abnormal *NOTCH2* structure. The analysis of the entire *NOTCH2* coding sequence was performed in four cases and did not reveal any mutation therefore indicating that *NOTCH2* does not harbor genetic characteristics of a tumor suppressor gene. Finally, the detailed analysis of chromosome 19 pericentromeric region led to the identification of two breakpoint clusters at 19p12 and 19q11–12. Interestingly, these two regions share a large stretch of homology. Together with previous observations of similarities between chromosome 1 and 19 alphoid sequences, this suggests that the t(1;19) translocation arises from complex intra and interchromosomal rearrangements.

This is the first comprehensive deletion mapping by high density oligo-array of the 1p/19q co-deletion in oligodendroglioma tumors using a methodological approach superior to others previously applied. As such this paper provides clear evidence that the *NOTCH2* gene is not physically rearranged by t(1;19) translocation of oligodendroglioma tumors.

## Introduction

Concurrent deletion of chromosomal arms 1p and 19q is the most common genetic alteration in oligodendroglial tumors [1]–[6]. Indeed, it is detected in 50% to 80% of oligodendroglial tumors including oligodendrogliomas (ODs) and oligoastrocytomas [7]–[9]. In contrast 1p/19q codeletion is uncommon in diffuse astrocytomas [3]. Combined 1p/19q allelic loss is observed in both grade II and grade III ODs. In patients affected with oligodendroglioma this genotype is associated with increased progression-free and overall survival as well as a better responsiveness to durable response to chemotherapy [4], [10], [11], [12]. We have previously constructed a 1 Mb resolution BAC array containing 3342 genomic clones covering the human genome and applied it to profile DNA copy number alterations of an extended series of 112 gliomas, including 49 ODs [13]. In the course of this study, we observed the presence of a consistent chromosome 1 breakpoint in the vicinity of the centromere in tumors presenting 1p/19q allelic loss. Based on this observation, we hypothesized that the break itself rather than the deletion might play a role in tumor development, supporting therefore the presence of a t(1;19) translocation. This hypothesis was further reinforced by studies of Jenkins et al. [14] and Griffin et al. [15] showing that an unbalanced t(1;19)(q10;p10) translocation accounts for the 1p/19q pattern and that this combined co-deletion results from the loss of one of the two translocation derivatives. Moreover, we and others [13], [16] found that 1p juxta-centromeric deletion breakpoints map within the region encoding the *NOTCH2* gene which is implicated in oligodendrocyte differentiation [17] and brain tumor oncogenesis [18].

*NOTCH2* constitutes a very attractive candidate to be specifically rearranged by the t(1;19) translocation. Rearrangement could lead to the formation of a fusion gene or to truncation of the gene. To more thoroughly investigate the *NOTCH2* region we constructed a high density, locus-specific oligonucleotide array-CGH covering the entire 1p pericentromeric region and applied it to profile 11 ODs with 1p/19q co-deletion. The chromosome 19 pericentromeric region was also investigated using the same approach. Finally, in order to further evaluate a potential tumor suppressor genetic model we searched for point mutations of the *NOTCH2* gene in four of our OD cases.

## Results

### *NOTCH2* as a candidate target of the pericentromeric t(1;19) translocation in oligodendrogliomas

**Figure 1:**
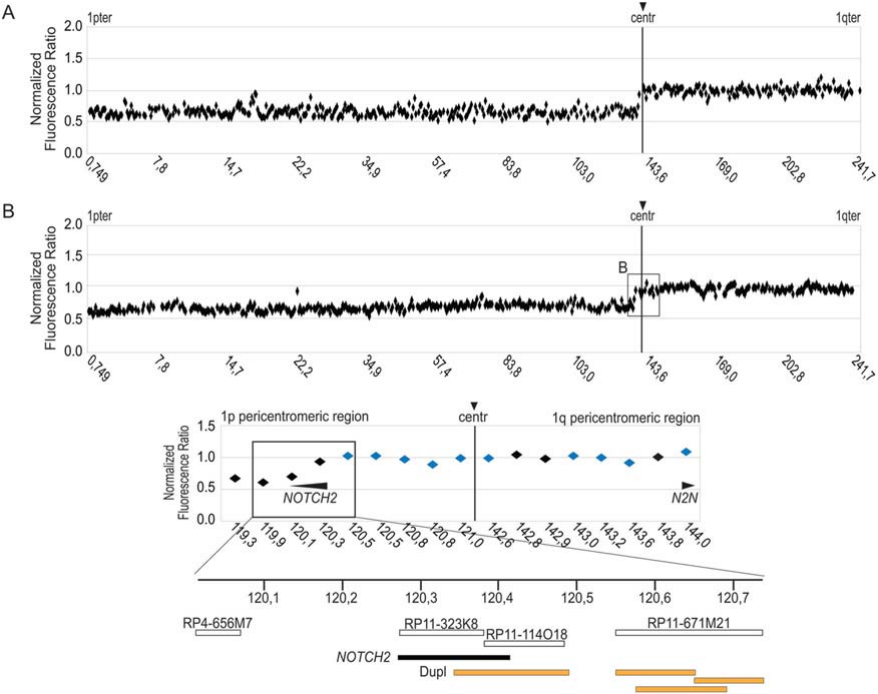
Detection of chromosome 1p recurrent deletions in oligodendrogliomas using 1 Mb BAC array-CGH. The vertical line denotes the centromere of chromosome (centr). The X-axis displays the physical position on chromosome 1 in megabases. A position landmark is indicated every 50 BACs. The Y-axis denotes the average normalized fluorescence ratio (ratio of means for the intensities between test/tumor and reference/normal DNA). (A) Array-CGH profiles illustrating 1p deletion in two selected oligodendrogliomas (OD16 and OD839) presenting 1p/19q co-deletion pattern. The box centers on the centromere and pericentromeric region shown in figure 1B. (B) Detailed view of 181 Kb recurrent deletion breakpoint segment. The blue diamonds highlight the supplementary clones in the improved version of a 1 Mb BAC array used in this analysis. Black arrows above the genes show the orientation of their transcription. The position of the distal end of each BAC is indicated. The lower part of the figure shows an enlargement of the box with indication of BAC clones (open bars), the *NOTCH2* gene (black bar) and segmental duplications (Dupl, orange bar).

Figure 1 displays chromosome 1 BAC-array results for two ODs DNAs, representative of the series of 49 ODs previously described [13]. In all cases, decreased tumor/normal fluorescence ratios, indicative of a deletion in the tumor DNA, were detected starting from BAC RP11-323K8 to the telomere of 1p (Figure 1). Conversely, normal fluorescence ratios were observed from BAC RP11-114O18 to the telomere of 1q. These results strongly suggested that a recurrent chromosome breakpoint, lying between BAC RP11-323K8 and BAC RP11-114O18 mapping at physical position 120,191,966–120,379,651 and 120,191,966–120,379,651 on chromosome 1, was a characteristic feature of ODs with 1p/19q co-deletion. Intriguingly, this region is included within *NOTCH2*. Given that the 1p/19q co-deletion pattern has been recently linked to a t(1;19) chromosome translocation, this suggested that the chromosome 1 breakpoint may lie within the *NOTCH2* gene. However, this putative breakpoint coincides with a transition from a single copy region (BAC RP11-323K8) to a segmental duplication (BAC RP11-114O18). Indeed, the sequence of this last BAC is duplicated at 1q21.1 (Figure 1B) which encodes a truncated copy of *NOTCH2*, NOTCH homolog 2 N-terminal like (*N2N*). Unlike common repeats (e.g. Alu or long interspersed nuclear elements (LINEs), the duplicated sequences in BAC clones resist complete blocking by human Cot-1 DNA, specifically when duplications are large and highly identical [19]. Therefore we could not conclude from these experiments whether the chromosome 1 breakpoint was localized within the *NOTCH2* gene or whether the apparent position of this break was falsely attributed to the presence of a segmental duplication.

### Exclusion of *NOTCH2* as candidate target of t(1p;19q) translocation–high density loci-specific oligonucleotide array

To investigate in depth the putative breakpoint regions we constructed a high density, oligonucleotide probes-based array (Agilent Technologies) providing high resolution over the pericentromeric regions of chromosome arms 1p and 19q. This loci-specific array was applied to profile OD DNAs derived from 15 patients. Each tumor DNA was paired with corresponding peripheral blood-derived DNA of the patient and with normal male DNA in individual experiments (used as references).

**Figure 2:**
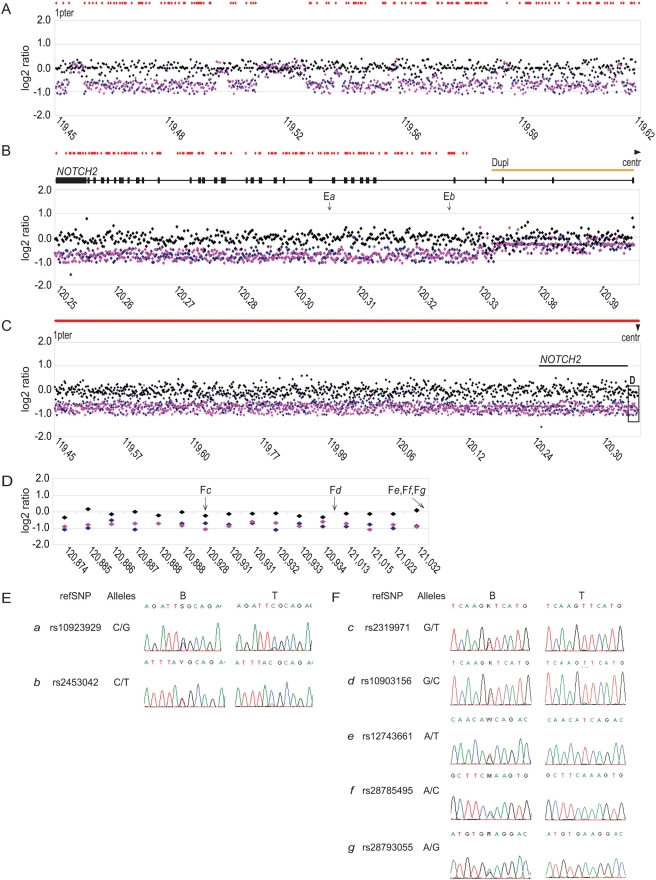
Deletion extends centromeric to *NOTCH2* as revealed by the high density oligonucleotide array. The X-axis displays the oligonucleotide probe order according to their physical position on chromosome 1 in megabases. A position landmark is indicated every 100 probes. The Y-axis denotes the average normalized fluorescence ratios on a log2 scale. Black diamonds represent the normalized log2 ratio from hybridization of patient *versus* reference (normal male) peripheral blood-derived DNAs, used as control experiment. The blue and pink diamonds illustrate normalized log2 ratios of the patient tumor DNA to the reference or to the matched peripheral blood-derived DNAs, respectively. The red diamonds above the graphs indicate the probes for which BLAT search displayed only a single chromosomal position within human genome with 100% sequence identity. (A) log2 ratio plot of a representative oligodendroglioma DNA (OD1786) displaying 500 oligonucleotide probes (119,457,515–119,619,961) telomeric to *NOTCH2* gene. (B) Array-CGH profile of OD1786 displaying probes encompassing the *NOTCH2* gene. The positions of the 34 exons (black horizontal bar) of *NOTCH2* and the segmental duplication (Dupl; orange horizontal bar) are highlighted above the array-CGH profile. Probes located within segmental duplication loci displayed quasi-normal fluorescence ratio, consistent with the duplication of this region to 1q21.1. (C) A pool of 851 single hit probes, including 128 data points covering *NOTCH2* gene is visualized. The majority of the probes displayed an average fluorescence ratio close to −1 (haploid level), consistent with the presence of a deletion. The position of *NOTCH2* gene is indicated by a black horizontal bar. The black box (D) highlights deletion of loci centromeric to *NOTCH2*. (D) Enlarged, detailed view of the normalized fluorescence log2 ratio for 16 unique loci enclosed by the chromosomal position 120,874,063-121,032,434, centromeric to *NOTCH2*. The X-axis displays every unique probe according to its physical position on chromosome 1. The vertical arrows indicate the position of the selected LOH (loss of heterozygosity)-associated SNP (single nucleotide polymorphism) loci analyzed. (E) LOH at loci distal to *NOTCH2*. Sequencing data comparing SNPs constitutional (B) and tumor DNA (T) from the same patient. In (*a*) and (*b*) examples of SNPs (rs1923929 and rs2453042, respectively) located within segment of *NOTCH2* gene. (F) Detection of LOH within five SNP loci (c–*g*) (rs2319971, rs10903156, rs12743661 rs28785495 and rs28793055, respectively) located centromeric to *NOTCH2* gene.

The 1.8 Mb synthesized 1p pericentromeric-specific region consisted of 4262 measurement points of varying repeat/duplication content including 478 probes covering the *NOTCH2* gene. As shown in Figure 2A the general distribution of aberrations defined by this high-resolution array supported the existence of 1p deletion in all studied cases. This figure displays output from the analysis of a representative case, illustrating a contiguous 165 kb region, telomeric to *NOTCH2* gene, (chromosome 1p position 119,457,515–119,622,059) covered by 500 probes. The probes fall into two major groups (Figure 2A, 2B). The first group mainly includes single copy probes, as assessed by a single hit through whole-genome BLAT analysis and indicated by red diamonds on the figure, which display average log2 fluorescence ratios close to −1 corresponding to a deletion profile. The second group exhibits normal (close to 0) log2 fluorescence ratios and mostly contains probes with multiple BLAT hits. As shown in figure 2B, the *NOTCH2* region can be divided in two segments. The 3′ part contains numerous single copy probes and presents a clear deleted profile whereas the 5′ duplicated end shows normal log2 ratios. These oligonucleotide array results are in perfect agreements with the BAC array data described above showing convergence of the apparent distal breakpoint of the deletion with the starting point of *NOTCH2* intrachromosomal duplication. In order to define reliable copy number changes we selected the probes for which the BLAT search displayed only a single chromosomal position within human genome with 100% sequence identity (probes indicated by red diamonds in Figure 2A). As a result, a group of 851 unique probes on chromosome 1, including 128 data points covering the *NOTCH2* gene, was extracted and is visualized on the graph (Figure 2C). Strikingly, 16 unique data points located centromeric of the *NOTCH2* gene enclosed by the chromosomal position 120,874,063–121,032,434 clearly indicated consistently decreased fluorescence ratios (Figure 2C, 2D) in all studied cases. To confirm that the deletion extended to the centromere, we further performed LOH-associated SNP loci analysis on five selected 1p/19q oligodendroglioma cases using a panel of 39 single copy SNP (single-nucleotide polymorphisms) markers encompassing the *NOTCH2* locus. This analysis showed that informative SNPs, being proximal, within or distal to *NOTCH2* exhibited loss-of-heterozygosity profiles. Figures 2E and 2F show representative results for SNPs intragenic (E*a* and E*b*) or proximal to *NOTCH2* part (F*c* to F*g*). All above outlined results, therefore, confirmed the presence of continuous 1p deletions extending all the way to the centromere and consequently ruled out the chromosome 1 translocation breakpoint being localized within the *NOTCH2* gene.

### Mutation analysis of the *NOTCH2* gene sequence

An hypothesis which may account for the localization of the t(1;19) translocation breakpoint proximal to *NOTCH2* may rely on a two-hit inactivation process with one allele of *NOTCH2* being inactivated by the deletion and the remaining one by point mutation. To test this hypothesis, we screened for somatic mutations the complete coding sequence of *NOTCH2* gene in four tumor and paired peripheral blood derived DNAs. The 34 exon sequences of this gene transcript span 9371 bp and encode a protein of 2555 amino acids. Sequence analyses of these genomic DNAs revealed no heterozygous mutation in either of the *NOTCH2* exons (data not shown).

### Two clusters of translocation breakpoints on chromosome 19

**Figure 3:**
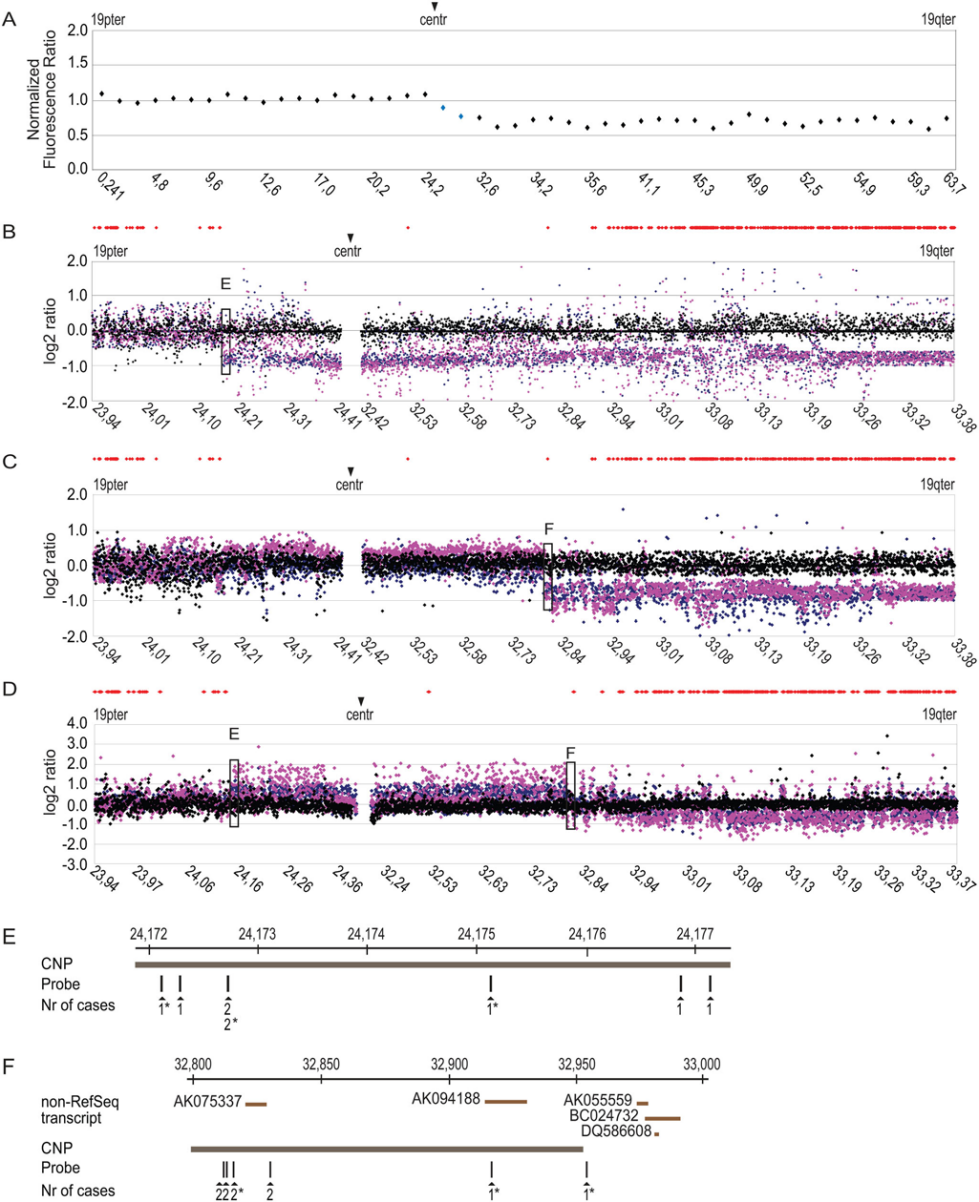
Array-CGH profiles of the representative cases containing deletions affecting chromosome 19. (A) Detection of deletion of the long arm of chromosome 19 using 1 Mb BAC array-CGH. The general outline of this figure follows Figure 1. The blue diamonds highlight the supplementary clones in the improved version of a 1 Mb BAC array used in this analysis. (B and C) Array-CGH profiles of two representative tumors disclosing the presence of continuous 19q deletion extending to: 19p (3B, OD1778) and 19q pericentromeric regions (3C, OD758). The positions of translocation breakpoints are highlighted by black boxes (E and F). The general outline of the figures follows Figure 2. (D) An example of oligodendroglioma case (OD1216) disclosing a gain which precedes 19q allelic loss. The positions of the breaks are highlighted by the black boxes (E and F). (E and F) Schematic representation of chromosome 19 breakpoint regions mapping at positions 24,172,000–24,177,100 and 32,800,000–33,000,000 within pericentromeric regions of chromosome 19. The oligonucleotide probes (vertical dark bars) are indicated according to their physical position on chromosome 19. The numbers of cases (Nr of cases) which display breakpoints are indicated. The cases displaying regional gain/amplifications are marked with an asterisk. The non-RefSeq transcripts (brown horizontal bar) and putative copy number polymorphisms (CNP) (gray horizontal bar) are drawn to scale.

As outlined above, the hemizygous deletion of the 19q arm (Figure 3A) strongly associates with 1p single copy loss, both events being mediated by a t(1;19) translocation with juxta-centromeric breakpoints. Therefore, we applied a chromosome 19 pericentromeric high density genomic microarray to precisely detect putative breakpoint sites. This array resolves the pericentromeric region of 1.4 Mb (positions on chromosome 19 23,940,099–24,422,759 and 32,426,023–33,387,097; build 36.1) into 3417 distinct measurement points with an average probe spacing of 379 bp. This 19q specific array proved to be a sensitive assay for detecting deletion of the 19q. In the course of these analyses three types of profile were retrieved. The first type consisted of a complete deletion of the 19q analyzed region extending down to chromosome 19p pericentromeric region. It was observed in 5/15 cases (Figure 3B). The second type, observed in 6/15 cases, was characterized by a transition from normal to deleted ratios within the analyzed 19q region (Figure 3C). The third class of profile, detected in 4 cases, revealed the coexistence of 19q loss with different patterns of gains/amplifications (Figure 3D). The positions of the breakpoints were slightly different from one case to the other (Figure 3E, 3F, for breakpoint positions information, see supplementary Table S1) and defined one major breakpoint cluster in 19p and another one on 19q. The 19p and 19q regions were determined to be 5 kb and 157 kb in size, respectively. The two cluster regions are shown on figure 3E and 3F. Using publicly available databases we reviewed the genes present in these regions and observed that only in the 19q region are there annotated gene sequences; namely the non-RefSeq transcripts AK075337, AK094188, AK055559, BC024732 and DQ586608. Both regions are also known to be encompassed by putative copy number polymorphism variation (CNP) (Figure 3E, 3F); however we did not find variation of these fragments in any of 15-germline DNA samples.

## Discussion

Our study follows up previous works that identified the existence of a nearly consistent deletion hotspot within pericentromeric region of 1p in ODs with 1p/19q allelic loss [13]. Interestingly, recent observations by Griffin et al. [15] and Jenkins et al. [14] also support the presence of a 1;19 translocation accompanied by a loss of one of the two translocation derivatives. The fact that *NOTCH2* is located near the chromosome 1 centromere made analysis of this interval a considerable challenge. Pericentromeres are among the most complex regions within human genome and contain several inter- and intra-chromosomal duplications and repeat sequences which increase in number when approaching the centromere [20]–[22]. The high density oligonucleotide array-CGH we applied is superior to previous methodological approaches used in the analysis of 1p/19q co-deleted OD [2], [10], [23], [24] with the major advantage being the high resolution of analysis due to the large number of independent measurement points completely covering *NOTCH2* gene and extending to the most centromeric point on the human reference sequence. We found that deletion on chromosomal arm 1p is in fact continuous and that its endpoint apparently lies within the centromere. Moreover, an additional detailed mutation analysis of all coding *NOTCH2* exons in four 1p/19q codeleted ODs presenting LOH showed no evidence of somatic *NOTCH2* mutations. This indicates that the deletion of one allele of *NOTCH2* is not associated with mutation of the other allele. Although haploinsufficiency and/or further genetic and epigenetic changes remain to be tested this indicates that *NOTCH2* does not present genetic characteristic of a tumor suppressor gene.

On chromosome 19 the high-resolution array-CGH analysis identified pericentromeric breakpoints in most cases. *In silico* study of 19q contrary to 19p pericentromeric region revealed the presence of highly repetitive architecture with multiple various classes of low copy repeat sequences such as Alpha, human satellite IV (HSAT4) and human endogenous retrovirus (HERVK) repeats at breakpoint sites (not shown). BLAT analysis of human sequence assembly showed intra- and interchromosomal sequence similarity of this locus. In particular it highlighted a high sequence homology between the two chromosome 19 cluster regions over a more than 50 kb stretch. We may therefore speculate that chromosome 19 pericentromeric loci comprise specific recombination-prone genomic structures leading to chromosomal instability by recombination-driven rearrangements. Importantly, strong DNA sequence homology of chromosome 19 to chromosome 1 centromeric and pericentromeric regions with several common alpha-satellite sequences was described [21], [25]. This suggests that the specific architecture of these segments might be more prone to chromosomal rearrangements, thus mediating chromosome breakage and generating the aforementioned 1p/19q translocation. Alphoid repeats have already been documented in some derivatives of the recurrent translocations suggesting genomic instability as a cause of arm-to-arm translocations in several types of tumors [26]–[28]. We cannot exclude other mechanisms that may be responsible for the profiles revealed by the array-CGH analysis of these 1p/19q co-deleted cases. Indeed, copy number polymorphisms encompassing the chromosome 19 breakpoint regions suggesting that some alternative structure of these regions may favor 1p/19q recurrent rearrangement in OD cases. It has been shown, for example, that complex repeat structures with functionally relevant polymorphism not only presents functional relevance to promoter activity but also demonstrate genetic instability in colorectal tumors of the mutator phenotype [29]. However, the peripheral blood versus reference DNA CGH experiments that were conducted as control experiments in our work did not identify obvious copy number variations. Finally, cancer-specific 1p/19q translocation might be facilitated by general genomic instability associated with alterations of the pericentromeric chromatin structure by epigenetic perturbation. Evidence suggests that the hypomethylation process induces pericentromeric heterochromatin decondensation and contributes to the instability of the satellite DNA sequences. This may permit recombination and formation of unstable translocations. Such decondensation has been described in glioblastoma as well as in other type of tumors such as facial anomalies syndrome, multiple myeloma, hepatocellular carcinomas, breast and Wilms tumors [30]–[36].

The data presented here revealed that chromosome 1 and 19 status in the tumors displaying t(1;19) recurrent translocations is more complex than previously thought. In summary, we exclude *NOTCH2* gene as a target of apparent translocation breakpoint and suggest involvement of the centromere and centromere/pericentromere sequences of chromosome 1 and 19, respectively, in the process of translocation. We also reveal novel pericentromeric breakpoint sites on 19p and 19q arms.

## Materials and Methods

### Clinical material

The current study consisted of oligodendroglioma tumor samples treated at the Pitié-Salpêtrière hospital, Paris, France and previously shown to harbor 1p/19q co-deletion [13]. Tumor samples were snap-frozen and stored at −80°C immediately after surgical resection. High molecular weight DNA was isolated from both tumor and peripheral blood using a standard phenol-chloroform procedure. All patients gave written informed consent, as requested by French law, allowing molecular, genetic and translational research studies on cancer tissue samples. The analysis was performed on anonymized data.

### 1 Mb genomic BAC ARRAY-CGH platform

A full-coverage genomic BAC aCGH with an average resolution of 1 Mb, previously described [11], was used for DNA copy number analysis. In particular, it contains a set of 402 measurement points localized on 1p, as well as 41 chromosome loci confined to chromosome 19. In order to increase the resolution of analysis an additional twenty-five BAC clones in the vicinity of the chromosome 1 centromere were further selected for inclusion on the initial 1 Mb array. The procedures for DNAs extraction, hybridization and washing have been described previously [13]. Arrays were scanned using a 4000B scan (Axon, Union city, USA). Image analysis was performed with Genepix 5.1 software (Axon, Union city, USA) and ratios of Cy5/Cy3 signals were determined.

### High density oligonucleotide array-CGH platform

The Agilent eArray platform was used to design oligonucleotide array-CGH (8×15 K, Agilent Technologies, Palo Alto, CA) providing high resolution over the pericentromeric regions on chromosome 1p 119,360,001–121,186,599, 1q 141,476,958–144,223,174, 19p 23,940,099–24,422,759 and 19q 32,426,023–33,387,097 of the Build 36.1 assembled human genome. In total, the pericentromeric-specific array consist of 4262 (1p), 4556 (1q), 1011 (19p) and 2406 (19q) measurement points of varying repeat/duplication content. In particular, total number of data points comprises 1901 single 100% sequence identity hit probes (128 covering *NOTCH2* gene) and these are abbreviated throughout the paper as unique probes. Finally, 1845 positive and negative biological control probes (localized on 1q and 19p and other human autosomes) were included into the array set-up. The designed array provides an average probe spacing of 377 bp. Sample amplification, labeling, and hybridization were performed according to the protocol provided by Agilent Technologies (8×15 K CGH oligonucleotide array-CGH array analyses). Briefly, 500 ng of genomic DNA was digested with AluI and RsaI enzymes followed by column purification (Microcon YM-30, Millipore, Valencia, CA, USA). Digested tumor and reference DNAs were labeled with Cy5-dUTP and Cy3-dUTP, respectively (Genomic DNA labeling Kit Plus, Agilent Technologies, Palo Alto, CA), pooled and hybridized as instructed in the manufacturers protocol. Agilent scanner was used to obtain ratios of Cy5/Cy3 signals from the targets localized on the array. Normalization (dye bias and background correction) and analysis of microarray data was performed using Agilent Feature Extraction (version 9.5.3 Agilent Technologies) and CGH Analytics software's (version 3.4.40, Agilent Technologies), respectively.

### Loss of heterozygosity analysis

To evaluate allelic loss, a panel of 39 and 42 SNPs (single-nucleotide polymorphisms) on 1p and 1q arm, respectively were studied. The locations and sequences from the LOH-associated SNP loci were retrieved from the database at the National Centre for Biotechnology Information (NCBI) (http://www.ncbi.nlm.nih.gov/SNP/). Primers flanking these loci were designed using Primer3. Oligonucleotide primer pairs for all SNPs were custom synthesized at MWG Biotech (http://www.the-mwg.com). Direct sequencing (Big Dye Terminator V3.1) of PCR-amplified locus fragments from blood and tumor DNA samples was carried out by 3130xl Genetic Analyser (Applied Biosystems, Inc.). Results obtained from the SNP loci in blood DNA were used as the baseline to estimate the copy number of the corresponding SNP loci in the tumor DNA. LOH was scored as present when the tumor sample showed reduction in height of one of two peaks (two alleles) by >50%. The consistency of all important results was validated by repeated analysis on both strands.

### Mutation analysis of the *NOTCH2* gene

All 34 *NOTCH2* encoding exons (Ensemble: ENSCAFG00000010476) of four selected oligodendroglioma tumor and paired peripheral blood derived DNAs were analyzed for somatic mutations by PCR amplification followed by direct genomic sequencing of both DNA strands (for additional information, see supplementary data, Table S2).

## Supporting Information

Table S1 (0.04 MB DOC)

Table S2 (0.07 MB DOC)
