# Supplementary material for: NOTCH2 Is Neither Rearranged nor Mutated in t(1;19) Positive Oligodendrogliomas
Source: PLoS One. 2009 Jan 1;4(1):e4107. doi: 10.1371/journal.pone.0004107 (PMC2606061; doi:10.1371/journal.pone.0004107)
Supplement: Table S1 — (0.04 MB DOC) [file pone.0004107.s001.doc]

**Table S1**. Summary of breakpoint positions on chromosome 19 detected by a high-density oligonucleotide microarray.
