# Supplementary material for: NOTCH2 Is Neither Rearranged nor Mutated in t(1;19) Positive Oligodendrogliomas
Source: PLoS One. 2009 Jan 1;4(1):e4107. doi: 10.1371/journal.pone.0004107 (PMC2606061; doi:10.1371/journal.pone.0004107)
Supplement: Table S2 — (0.07 MB DOC) [file pone.0004107.s002.doc]

**Table S2**. List of primer sequences used for *NOTCH2* mutation analysis
